# Repeated predictable stress causes resilience against colitis-induced behavioral changes in mice

**DOI:** 10.3389/fnbeh.2014.00386

**Published:** 2014-11-06

**Authors:** Ahmed M. Hassan, Piyush Jain, Florian Reichmann, Raphaela Mayerhofer, Aitak Farzi, Rufina Schuligoi, Peter Holzer

**Affiliations:** Research Unit of Translational Neurogastroenterology, Institute of Experimental and Clinical Pharmacology, Medical University of GrazGraz, Austria

**Keywords:** DSS-induced colitis, resilience, anxiety, social interaction, cyclooxygenase 2, neuropeptide Y, corticosterone, gut-brain axis

## Abstract

Inflammatory bowel disease is associated with an increased risk of mental disorders and can be exacerbated by stress. In this study which was performed with male 10-week old C57Bl/6N mice, we used dextran sulfate sodium (DSS)-induced colitis to evaluate behavioral changes caused by intestinal inflammation, to assess the interaction between repeated psychological stress (water avoidance stress, WAS) and colitis in modifying behavior, and to analyze neurochemical correlates of this interaction. A 7-day treatment with DSS (2% in drinking water) decreased locomotion and enhanced anxiety-like behavior in the open field test and reduced social interaction. Repeated exposure to WAS for 7 days had little influence on behavior but prevented the DSS-induced behavioral disturbances in the open field and SI tests. In contrast, repeated WAS did not modify colon length, colonic myeloperoxidase content and circulating proinflammatory cytokines, parameters used to assess colitis severity. DSS-induced colitis was associated with an increase in circulating neuropeptide Y (NPY), a rise in the hypothalamic expression of cyclooxygenase-2 mRNA and a decrease in the hippocampal expression of NPY mRNA, brain-derived neurotrophic factor mRNA and mineralocorticoid receptor mRNA. Repeated WAS significantly decreased the relative expression of corticotropin-releasing factor mRNA in the hippocampus. The effect of repeated WAS to blunt the DSS-evoked behavioral disturbances was associated with a rise of circulating corticosterone and an increase in the expression of hypothalamic NPY mRNA. These results show that experimental colitis leads to a particular range of behavioral alterations which can be prevented by repeated WAS, a model of predictable chronic stress, while the severity of colitis remains unabated. We conclude that the mechanisms underlying the resilience effect of repeated WAS involves hypothalamic NPY and the hypothalamic-pituitary-adrenal axis.

## Introduction

Inflammatory bowel disease (IBD) is a major health problem mainly in the Western countries where the prevalence of IBD is more than 200 per 100,000 inhabitants (Cosnes et al., 2011). The disease reduces quality of life, and the deterioration of well-being is exacerbated in the presence of psychiatric disturbances (Guthrie et al., 2002; Nordin et al., 2002). Several mental disorders including major depression, panic and generalized anxiety are more common in IBD patients than in community controls (Walker et al., 2008; Graff et al., 2009). Moreover, psychiatric disorders can affect disease prognosis and response to treatment. For example, infliximab is less efficacious in Crohns' disease patients with major depression than in non-depressed Crohn's disease subjects (Persoons et al., 2005). The relationship between IBD and changes in the brain is emphasized by epidemiological studies showing that stressful life events can be a risk factor for IBD development and relapse (Mawdsley and Rampton, 2006). The continuously accumulating evidence that connects IBD with psychiatric disorders underscores the necessity to study brain reactions to gastrointestinal inflammation.

Adding dextran sulfate sodium (DSS) to rodents' drinking water leads to the development of colitis. Showing many similarities to human IBD pathology, DSS-induced colitis is widely perceived as a model of IBD with good translational power (Solomon et al., 2010). Inspired by human data, the effect of psychological stress has repeatedly been tested in the DSS model of IBD and in most studies found to aggravate the severity of inflammation (Milde and Murison, 2002; Reber et al., 2006). Moreover, mice with DSS-induced colitis have been reported to exhibit anxiety-like behavior (Bercik et al., 2011; Painsipp et al., 2011), but the neurochemical basis of these behavioral alterations has not yet been addressed.

The molecular basis of behavioral changes associated with DSS-induced colitis has not yet been elucidated. Studies of other peripheral immune challenge models suggest that a concurrent inflammatory process in the brain may play a role. Peripheral injection of lipopolysaccharide (LPS) is associated with increased expression of cyclooxygenase 2 (COX-2), but not COX-1, in the hypothalamus (Cover et al., 2001). COX-2 and one of its products, prostaglandin E_2_, have been involved in LPS- and interleukin (IL)1β-induced anorexia which is inhibited by COX-2 but not COX-1 inhibitors (Lugarini et al., 2002; Asarian and Langhans, 2010). These findings raise the question whether COX-2 participates in the behavioral changes caused by DSS-induced colitis.

Psychological stress is a risk factor for several psychiatric disturbances. Stressful life events precede episodes of several mental disorders including generalized anxiety disorder and major depression (Blazer et al., 1987; Kendler et al., 2003). Water avoidance stress (WAS) is a model of psychological stress in rodents, in which animals are placed on a platform surrounded by water which prevents them from escaping. WAS efficiently stimulates stress-related circuits in the brain in rats and mice, and repeated exposure to WAS leads to anxiety-like behavior and visceral hyperalgesia in rats (Bonaz and Taché, 1994; Bradesi et al., 2005; Reichmann et al., 2013; Ait-Belgnaoui et al., 2014). However, repeated psychological stress can lead to a wide range of behavioral manifestations, occasionally being without effect (Gregus et al., 2005) or even improving behavioral performance. For example, 5-min sessions of restraint stress for 28 days improve mood, hippocampal neurogenesis, and cognitive function in rats (Parihar et al., 2011). Moreover, if applied in adolescence, the same stress protocol induces resilience against chronic unpredictable stress in adulthood (Suo et al., 2013). Despite the extensive literature that evaluated the influence of psychological stress on chemically induced colitis, very little is known about the effect of psychological stress on colitis-associated behavioral and molecular changes and whether or not psychological stress impacts on DSS-induced behavioral alterations.

The objective of this work, therefore, was to evaluate the interaction between repeated psychological stress (WAS) and DSS-induced colitis in modifying emotional-affective behavior and to analyze potential mechanisms behind this interaction. The study set out to address four specific aims. The first aim was to evaluate whether DSS-induced colitis in C57Bl6/N mice is associated with anxiety-like behavior, social isolation, and despair behavior. The second aim was to assess whether combining WAS with DSS would influence DSS-induced colitis and DSS-induced behavioral changes in a similar or differential manner. The third aim was to examine whether colonic inflammation extended to the brain by evaluating gene expression of cyclooxygenase 1 (COX-1) and cyclooxygenase 2 (COX-2) in the brain. Finally, the fourth aim was to explore the mechanistic basis of the interaction between stress and inflammation on brain function and behavior. This goal was addressed by investigating the plasma level of corticosterone and the cerebral expression of corticotropin-releasing factor (CRF), mineralocorticoid receptors (MR) and glucocorticoid receptors (GR) as important factors of the hypothalamic-pituitary-adrenal (HPA) axis and brain-derived neurotrophic factor (BDNF) and neuropeptide Y (NPY) as messengers relevant to emotional-affective behavior.

## Material and methods

### Experimental animals

The experiments were carried out with male C57BL/6N mice obtained from Charles River (Sulzfeld, Germany) at the age of 10 weeks. The animals were housed two per cage under controlled conditions of temperature (set point 21°C) and air humidity (set point 50%) and under a 12 h light/dark cycle (lights on at 6:00 h, lights off at 18:00 h). Standard laboratory chow was provided *ad libitum* throughout the study. The mice were habituated in the animal facility for 2 weeks before any intervention. All experiments were approved by an ethical committee at the Federal Ministry of Science, Research and Economy of the Republic of Austria (BMWF-66.010/0118-II/3b/2011 and BMWFW-66.010/0054-WF/II/3b/2014) and conducted according to the Directive of the European Communities Council of 24 November 1986 (86/609/EEC) and the Directive of the European Parliament and of the Council of 22 September 2010 (2010/63/EU). The experiments were designed in such a way that both the number of animals used and their suffering was minimized.

### Study design

In order to investigate the interaction between DSS-induced colitis and repeated WAS on behavioral changes, 68 mice were allocated to 4 experimental groups:

1. a control group (*n* = 16), handled once daily from day 1 to day 7,
2. the water avoidance stress (WAS) group (*n* = 16), exposed to intermittent WAS once daily for 1 h from day 1 to day 7,
3. the DSS colitis group (*n* = 18), receiving DSS (2%) in the drinking water and handled once daily from day 1 to day 7, and
4. the WAS+DSS group (*n* = 18), subjected to both WAS and DSS treatment from day 1 to day 7.

In study 1, the effect of a 7-day treatment with WAS, DSS, and WAS+DSS on the behavior of the animals was evaluated. Body weight was measured on day 1 and day 8. Anxiety-like behavior and locomotor activity were assessed with the open (OF) field test on day 8. On day 9, social activity was evaluated with the social interaction (SI) test. On day 10, depression-like behavior was evaluated with the tail suspension test (TST). On day 11 the mice were sacrificed by decapitation after they had been deeply anesthetized with pentobarbital (150 mg/kg IP) to collect the colon for myeloperoxidase (MPO) determination.

In study 2, the effect of a 7-day treatment with WAS, DSS, and WAS+DSS on molecular factors in the colon, blood, and brain was assessed in the absence of any behavioral tests. In this study the daily food and daily water intake were assessed by weighing the food pellets and the water bottles of the housing cages at the beginning of the WAS session. On day 8, the animals were sacrificed as described, and plasma, colon and brain collected. The total number of mice used in study 2 was 32 (*n* = 8 per group).

In both studies, all behavioral tests, plasma and tissue collections were carried out between 8:00 and 13:00 h.

### Induction of colitis

Mild colitis was induced by adding DSS (molecular weight 36,000–50,000; MP Biomedicals, Illkirch, France) at a concentration of 2% (w/v) to the drinking water for 7 days (Mitrovic et al., 2010). Control animals received normal tap water.

### Water avoidance stress

Mice were placed on a small platform (6 × 3 × 3 cm, length × width × height) in the center of a water-filled tank (50 × 32 × 30 cm, length × width × height), the level of the water in the tank being 0.5–1 cm below the platform. After a 60 min stay on the platform the animals were returned to their home cages (Melgar et al., 2008). The mice were not pre-trained to avoid jumping into the water. During the WAS session the mice were surveyed by an investigator. If mice jumped into the water, the investigator put them back immediately on the platform. It was observed that mice tried to escape more frequently in the first 2 days of the 7-day treatment period, whereas in the remaining 5 days the trials to escape were uncommon. The WAS procedure was carried out between 10:00 and 13:00 h. Each individual mouse was exposed to WAS every day at the same time of the day that did not vary more than 30 min.

### Open field test

The OF consisted of an opaque gray plastic box (50 × 50 × 50 cm, length × width × height). The ground area of the box was divided into a 36 × 36 cm central area and the surrounding border zone. The mice were placed individually in a corner of the OF, and their behavior during a 5 min test period was tracked by a video camera positioned above the center of the OF and recorded with the software VideoMot2 (TSE Systems, Bad Homburg, Germany). This software was used to evaluate the time spent in the central area, the number of entries into the central area, the total distance traveled in the OF, and the distance traveled in the central area. A reduction of the central area time, of the distance traveled in the central area, and/or of the central area entries was interpreted as an increase in anxiety-like behavior (Bailey and Crawley, 2009). The OF box was cleaned with water after each mouse had been tested.

### Social interaction test

The SI test was performed in the OF box as described (Tabuchi et al., 2007; de Theije et al., 2014). An empty cylindrical meshwork container (7 × 10 cm, diameter × height) was placed adjacent to the middle of one wall of the OF. The test mouse was placed adjacent to the middle of the opposite wall of the OF and allowed to explore the field for 3 min, after which the mouse was returned to its home cage. A novel mouse (target mouse) was placed in the cylindrical container, and then the test mouse was allowed to explore the OF for another 3 min. The time spent in the interaction zone which was within 8 cm of the cylindrical container was calculated with the VideoMot2 software in both sessions. Social activity was expressed as SI percent which was defined as the percent ratio between the time spent in the interaction zone in the presence of the target mouse divided by the time spent in the interaction zone in the absence of the target mouse. The OF box was cleaned with 70% ethanol after each mouse had been tested (de Theije et al., 2014).

### Tail suspension test

The animals were suspended by their tail with a 1.9 cm wide strapping tape (Leukotape classic, BSN Medical S.A.S., Le Mans, France) for 6 min, and their behavior was recorded by a video camera. A trained blinded observer analyzed the video recordings with the VideoMot2 software event monitoring module for 3 types of behavior: swinging, curling and immobility. The mouse was considered swinging when it continuously moved its paws while keeping the body straight and/or moving the body from side to side. The mouse was considered curling when the mouse twisted its trunk (Berrocoso et al., 2013). The time spent swinging, curling and being immobile was calculated. Mice which climbed over their tails were excluded as they had learned that escape is possible (Cryan et al., 2005).

### Collection of blood and tissues

Blood was collected by cardiac puncture with 3.8% citrate as anticoagulant during sacrifice within 3.5 min after injection of pentobarbital. After centrifugation at 1600 × g for 15 min at 4°C, the plasma was frozen immediately on dry ice and stored at −70°C until assay.

Colon length and colon weight were used to assess colitis severity (Vowinkel et al., 2004). After decapitation of the mice, the colon extending from the proximal end at the cecum to the anal end was rapidly removed and its length measured. Subsequently the colon was opened longitudinally, washed under running water, dried with tissue paper, and its weight (mg) determined. Then the distal part of the colon was shock-frozen in liquid nitrogen and stored at −70°C until MPO assay. The brains were removed, frozen on dry ice, wrapped in aluminum foil, and stored at −70°C.

### Colonic myeloperoxidase measurement

MPO is an enzyme found mainly in neutrophils, monocytes and macrophages and has frequently been used to quantify experimental colitis severity (Krawisz et al., 1984). The MPO content of the colon was measured with an enzyme-linked immunosorbent assay kit specific for the rat and mouse protein (Hycult Biotechnology, Uden, The Netherlands). The tissue samples were prepared according to the manufacturer's instructions. After weighing, the frozen tissues were placed in MPO lysis buffer (pH 7.4) at a ratio of 1 mg: 0.02 ml. The composition of the lysis buffer was: 200 mM NaCl, 5 mM ethylenediaminetetraacetic acid, 10 mM trishydroxy methylaminomethane, 10% glycerine, 1 mM phenylmethylsulphonyl fluoride, 1 mg/ml leupeptide, and 28 mg/ml aprotinin.

The samples were homogenized on ice with an Ultraturrax (IKA, Staufen, Germany) and then subjected to two centrifugation steps at 6000 × g and 4°C for 15 min. The MPO content of the supernatant was measured with the kit. The sensitivity of the assay was 1 ng/ml at an intra- and inter-assay variation of around 10%.

### Multiplex measurement of plasma cytokines

The plasma levels of IL-6, IL-10, IL-12, and IL-18 were measured with a multiplex immunoassay (ProcartaPlex Multiplex Immunoassays, eBioscience, Vienna, Austria). The assay was performed according to the manufacturer's instructions. The fluorescent signal was measured with the Bio-Plex 200 multiplex suspension array system in combination with the Bio-Plex 5.0 Software (Bio-Rad, Hercules, CA, USA). Standard curves were generated with a five-parameter logistic curve-fitting method. Cytokines that were below detection limit were assigned a value of zero. The sensitivities of the assay were 0.9, 0.35, 0.35, and 8.09 pg/ml for IL-6, IL-10, IL-12, and IL-18, respectively.

### Plasma neuropeptide Y (NPY) and corticosterone measurements

The plasma levels of NPY and corticosterone were determined with specific enzyme immunoassay kits according to the manufacturer's instructions. The sensitivity of the NPY kit (Phoenix Pharmaceuticals, Burlingame, CA, USA) was 0.09 ng/ml, while the sensitivity of the corticosterone kit (Assay Designs, Ann Arbor, MI, USA) was 0.027 ng/ml.

### Brain microdissection

For the microdissection procedure, the frozen brains were transferred to a cryostat at −20°C and cut manually into approximately 1 mm thick slices. These brain slices were placed on a cold plate (Weinkauf Medizintechnik, Forchheim, Germany) set at −20°C, on which hypothalamus, amygdale, and hippocampus were microdissected under a stereomicroscope. Hypothalamic tissue was collected from the preoptic area (Bregma: +0.26) to the end of the mammillary bodies (Bregma: −2.92), amygdalar tissue from the anterior edge of the optical tract (Bregma: −0.58) to the posterior part of the basolateral and basomedial amygdala (Bregma: −2.54), and hippocampal tissue from the limit of the hippocampal formation (Bregma: −0.94) to the caudal end of the dentate gyrus (Bregma: −4.04). The microdissected brain areas were kept in homogenization tubes on dry ice and subsequently stored at −70°C until further processing (Brunner et al., 2014).

### RNA extraction and real time PCR

RNA was extracted from the microdissected brain regions with the RNeasy Lipid Tissue Mini Kit (Qiagen, Hilden, Germany). Aliquots of 1 μg RNA were reverse-transcribed with the High Capacity cDNA Reverse Transcription kit (Applied Biosystems, Foster City, CA, USA).

For relative quantification of mRNA, real time PCR was performed with the CFX Connect™ Real-Time PCR detection system in combination with the CFX Manager™ software 3.1 (Bio-Rad). The specific primers used for amplification and quantitation of mRNA are listed in Table 1. GAPDH (Mm_Gapdh_3_SG QuantiTect Primer Assay, Qiagen) was used as reference gene. The stability of GAPDH as a reference gene was confirmed with the M value (Vandesompele et al., 2002; Hellemans et al., 2007) as assessed by the CFX Manager™ 3.1 software. The PCR SsoAdvanced™ Universal SYBR® Green Supermix (Bio-Rad) was used for amplification, and the cycling conditions were as follows: samples were heated to 95°C for 30 s followed by 39 cycles of 95°C for 3 s, and 60°C for 30 s. Except for the GAPDH primers which have been validated by the manufacturer, the products of all other primers were sequenced to confirm specificity. Quantitative values of mRNA relative to control were calculated with the 2^−ΔΔ CT^ method (Schmittgen and Livak, 2008).

**Table 1:** **Primers used in the study**.

**Gene**	**Primer sequence (5′→3′)**	**References**
BDNF forward	GTGACAGTATTAGCGAGTGG	Designed by Primer-Blast (Ye et al., 2012)
BDNF reverse	TTCTCTAGGACTGTGACCGT	
COX-1 forward	ATGAGTCGAAGGAGTCTCTCG	Harvard PrimerBank (Spandidos et al., 2010) ID: 6679537a1
COX-1 reverse	GCACGGATAGTAACAACAGGGA	
COX-2 forward	TTCAACACACTCTATCACTGGC	Harvard PrimerBank ID: 31127110a1
COX-2 reverse	AGAAGCGTTTGCGGTACTCAT	
CRF forward	GAATTTCTTGCAGCCGGAGC	Designed by Primer-Blast
CRF reverse	CAGCGGGACTTCTGTTGAGA	
GR forward	GACTCCAAAGAATCCTTAGCTCC	Harvard PrimerBank ID: 121247452c1
GR reverse	CTCCACCCCTCAGGGTTTTAT	
MR forward	GAAGAGCCCCTCTGTTTGCAG	Harvard PrimerBank ID: 17384011a1
MR reverse	TCCTTGAGTGATGGGACTGTG	
NPY forward	CAGATACTACTCCGCTCTGCGAC ACTACAT	Ferenczi et al., 2010
NPY reverse	TTCCTTCATTAAGAGGTCTGAAAT CAGTGTCT	

### Statistics

SPSS 21 and SigmaPlot 12.1 were used for statistical analysis and graphic presentation of the results. The data were analyzed with Two-Way ANOVA, one factor being DSS treatment, and the other factor being WAS. Log transformation was considered whenever needed to meet Two-Way ANOVA assumptions. For the analysis of colonic MPO levels ANOVA was performed after rank transformation. Whenever a significant interaction between WAS and DSS was found, a Bonferroni post-hoc test was carried out. Main factor effects and interactions between the two factors were considered significant if *p* ≤ 0.05. Daily food and water intake was evaluated with repeated measures ANOVA. Sphericity assumptions were checked by Mauchly's test and, in case of violation of sphericity, the Greenhouse-Geisser correction was used. Since the levels of proinflammatory cytokines did not meet Two-Way ANOVA assumptions, the Kruskal-Wallis test was employed and post-hoc the Mann-Witney U test with Bonferroni correction was used for pairwise comparisons.

## Results

### WAS had no major effect on the severity of DSS-induced colitis

**Figure 1:**
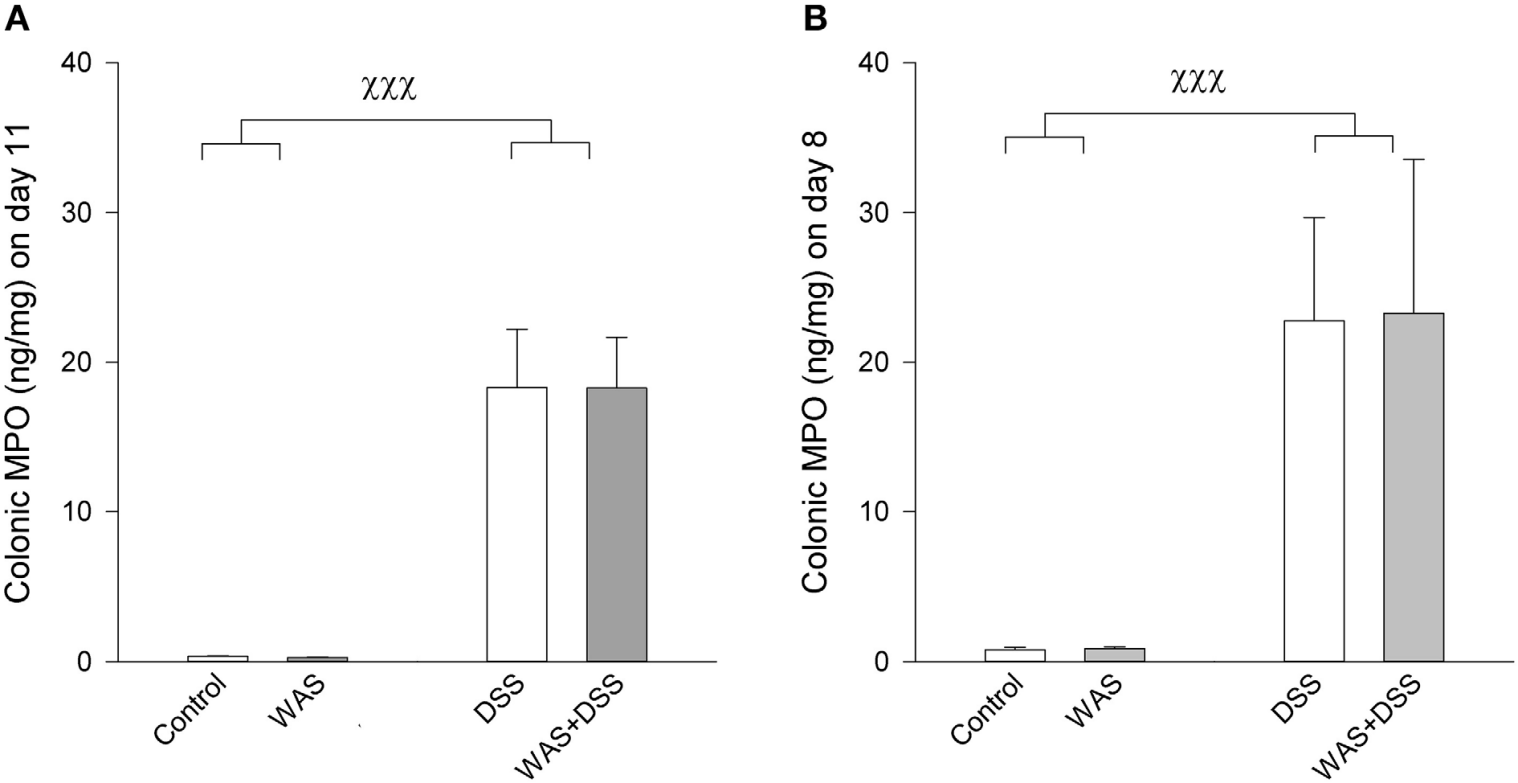
**Colonic MPO content in mice subjected to a 7-day treatment with WAS, DSS, or WAS+DSS (*n* = 15–18 per group) followed by behavioral testing during days 8–10 as carried out in study 1 (A) or sacrificed on day 8 (*n* = 8 per group) without behavioral testing as examined in study 2 (B)**. In both studies Two-Way ANOVA disclosed a significant DSS effect (*p* < 0.001) but no WAS effect and no significant interaction. The data shown are means + s.e.m., ^χχχ^*p* < 0.001 DSS main factor effect.

Treatment with 2% DSS induced colitis, the severity of which was assessed by animal weight, colon length, colon weight (Tables 2, 3), and colonic MPO levels (Figure 1). As shown in Tables 2, 3, DSS treatment caused body weight loss, increased colon weight and reduced colon length. These changes were also seen in animals treated with WAS+DSS but were absent in mice treated with WAS alone. Two-Way ANOVA in the animals subjected to behavioral tests (study 1, Table 2) revealed a significant main factor effect of DSS treatment on weight loss [*F*_(1, 64)_ = 55.7; *p* < 0.001], colon weight [*F*_(1, 64)_ = 148.2; *p* < 0.001], and colon length [*F*_(1, 64)_ = 182.3; *p* < 0.001]. In none of the experimental groups was there any significant interaction between the factors WAS and DSS (Table 2). In addition, DSS treatment led to a significant rise of the colonic MPO content [main factor effect: *F*_(1, 62)_ = 187; *p* < 0.001] while WAS had no significant effect and there was no significant interaction between WAS and DSS (Figure 1A).

**Table 2:** **Effect of DSS and WAS, alone and in combination on body weight loss, colon weight, and colon length in mice that underwent behavioral tests**.

	**Control *n* = 16**	**WAS *n* = 16**	**DSS *n* = 18**	**WAS + DSS *n* = 18**	**WAS main factor effect**	**DSS main factor effect**	**Interaction**
Body weight change (g) on day 8	+ 0.31 (0.18)	−0.06 (0.28)	−1.39 (0.22)	−1.83 (0.28)	NS	*p* < 0.001	NS
Colon weight (mg/cm) on day 11	29.9 (0.86)	31.7 (0.97)	49.6 (1.99)	47.7 (1.54)	NS	*p* < 0.001	NS
Colon length (cm) on day 11	7.80 (0.19)	7.63 (0.13)	5.81 (0.15)	5.51 (0.14)	NS	*p* < 0.001	NS

The mice were sacrificed 4 days after the end of treatment with DSS and WAS.

Animals were subjected to a 7-day treatment (days 1–7) with DSS (2% in the drinking water) and WAS (1 h daily), alone or in combination, followed by behavioral testing during days 8–10. The mice were sacrificed on day 11, i.e., 4 days after the end of treatment with DSS and WAS. The data are presented as means (s.e.m.). NS, not significant.

**Table 3:** **Effect of DSS and WAS, alone and in combination, on body weight loss, colon weight, and colon length in mice used for the assay of biochemical factors in the colon, blood and brain in the absence of any behavioral testing**.

	**Control *n* = 8**	**WAS *n* = 8**	**DSS *n* = 8**	**WAS + DSS *n* = 8**	**WAS main factor effect**	**DSS main factor effect**	**Interaction**
Body weight change (g) on day 8	+ 0.64 (0.17)	+ 0.02 (0.17)	−2.47 (0.29)	−3.90 (0.57)	*p* < 0.01	*p* < 0.001	NS
Colon weight (mg/cm) on day 8	30.9 (1.49)	28.6 (0.76)	41.7 (1.71) ^***^	46.7 (1.87) ^§^^###^	NS	*p* < 0.001	*p* < 0.05
Colon length (cm) on day 8	8.65 (0.35)	8.75 (0.27)	6.51 (0.40)	5.95 (0.30)	NS	*p* < 0.001	NS

The mice were sacrificed 1 day after the end of treatment with DSS and WAS.

*Animals were subjected to a 7-day treatment (days 1–7) with DSS (2% in the drinking water) and WAS (1 h daily), alone or in combination, and sacrificed for the assay of biochemical factors in the colon, blood and brain on day 8, i.e., 1 day after the end of treatment with DSS and WAS. The data are presented as means (s.e.m.)*.

*** *p* < 0.001 vs. control (colon weight),

§ *p* < 0.05 vs. DSS (colon weight),

### *p* < 0.001 vs. WAS (colon weight). NS, not significant.

In the animals that were used for the assay of biochemical factors in the colon, blood and brain in the absence of any behavioral testing (study 2), DSS had a similar effect as in study 1 (compare Tables 2, 3). Two-Way ANOVA showed a significant main factor effect of DSS on body weight loss [*F*_(1, 28)_ = 106.8; *p* < 0.001], and colon length [*F*_(1, 28)_ = 54.7; *p* < 0.001]. A significant main factor effect of WAS was observed only with body weight loss [*F*_(1, 28)_ = 9.2; *p* = 0.005], and a significant interaction between the factors WAS and DSS was disclosed for colon weight only [*F*_(1, 28)_ = 5.7; *p* = 0.024]. *Post-hoc* analysis revealed that DSS significantly increased colon weight, an effect that was exacerbated by WAS while WAS alone did not affect colon weight (Table 3). The colonic MPO content was significantly elevated by DSS [main factor effect: *F*_(1, 28)_ = 86.7; *p* < 0.001] but not WAS, without any significant interaction between the two factors (Figure 1B).

### DSS, but not WAS, reduced the daily food intake

**Figure 2:**
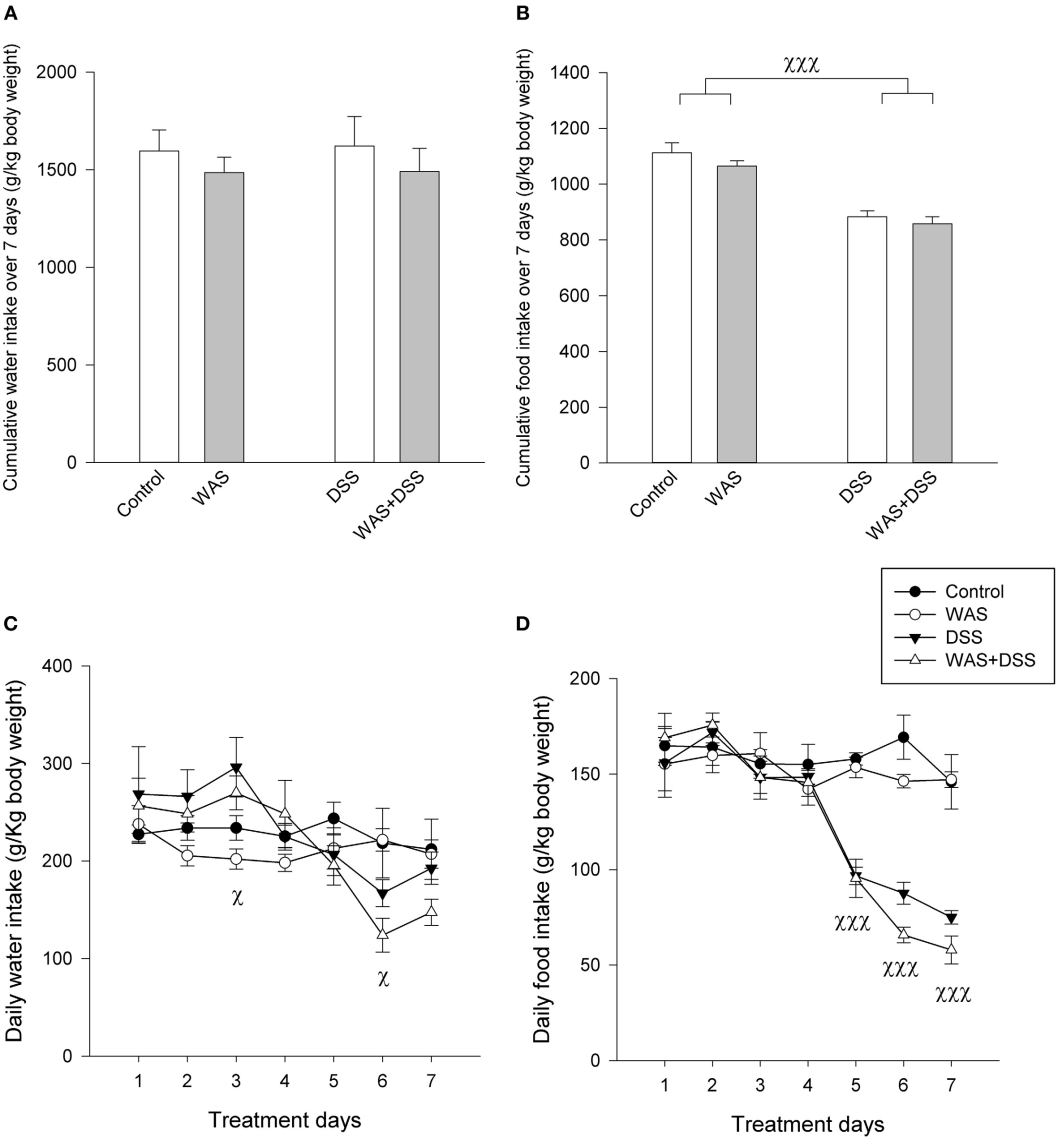
**Effect of a 7-day treatment with DSS and WAS, alone and in combination, on water and food intake as examined in study 2**. **(A)** Cumulative water intake over the 7-day treatment period. There were no significant differences. **(B)** Cumulative food intake over the 7-day treatment period. There was a significant main factor effect of DSS on total food intake (*p* < 0.001). **(C)** Daily water consumption during the 7-day treatment period. Repeated measures ANOVA disclosed that DSS significantly modified the daily water intake during the 7-day observation period. *Post-hoc* testing showed a significant main factor effect of DSS on days 3 and 6 (*p* < 0.05). **(D)** Daily food consumption during the 7-day treatment period. Repeated measures ANOVA revealed that DSS significantly suppressed the daily water intake. *Post-hoc* testing showed a significant main factor effect of DSS during days 5–7 (*p* < 0.001). The data shown are means + s.e.m. **(A,B)** and means ± s.e.m. **(C,D)**, *n* = 4 cages per group each housing two mice, ^χ^*p* < 0.05 DSS main factor effect, ^χχχ^*p* < 0.001 DSS main factor effect.

The cumulative water intake over the 7-day treatment period was not significantly changed in response to the WAS and DSS treatments (Figure 2A). Repeated measures ANOVA (Figure 2C), however, showed a significant change of the daily water intake during the 7-day treatment period [*F*_(6, 13)_ = 13.1; *p* < 0.001], with a significant interaction with DSS [*F*_(6, 13)_ = 11.1; *p* < 0.001] but not WAS. In the DSS-treated mice, the daily water intake was enhanced during days 1–4 but this difference was statistically significant only on day 3 (Figure 2C). Subsequently, the daily water intake dropped below the daily water intake of control mice, this difference being statistically significant on day 6 (Figure 2C).

Food intake was more prominently modified by the 7-day treatment with DSS (Figures 2B,D) than water intake. Two-Way ANOVA showed that the cumulative intake over the 7-day treatment period was significantly reduced in response to DSS [*F*_(1, 12)_ = 68.7; *p* < 0.001] but not WAS (Figure 2B), without a significant interaction between the two factors. Repeated measures ANOVA revealed a significant reduction of the daily food intake (Figure 2D) during the 7-day treatment period [*F*_(2.6, 31.2)_ = 26.8; *p* < 0.001] with a significant interaction with DSS, but not WAS [*F*_(2.6, 31.2)_ = 20.3; *p* < 0.001]. *Post-hoc* analysis disclosed that the daily food intake of DSS-treated mice was significantly attenuated during days 5–7 (Figure 2D).

### WAS prevented the DSS-induced behavioral changes in the OF and SI tests but not in the TST

**Figure 3:**
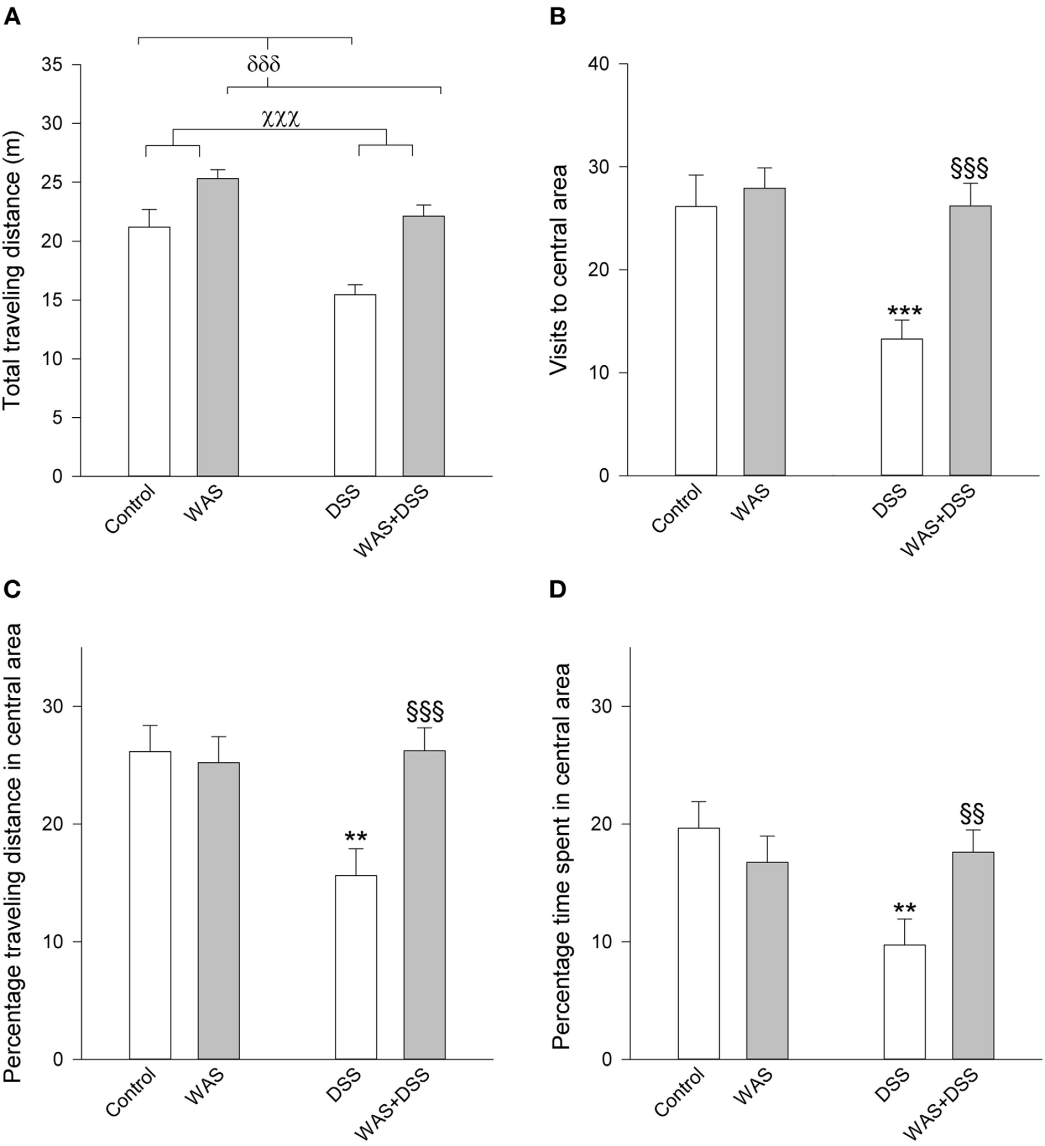
**Effect of a 7-day treatment with DSS and WAS, alone and in combination, on behavioral parameters of the OF test as carried out 1 day after the end of the treatment period**. **(A)** Total traveling distance. Both WAS and DSS had a main factor effect (*p* < 0.001), but there was no significant interaction between the two factors. **(B)** Visits to central area. There was a significant interaction between WAS and DSS in this parameter (*p* < 0.05). **(C)** Percentage traveling distance in central area, calculated as a percentage of the total traveling distance. There was a significant interaction between WAS and DSS in modifying this parameter (*p* < 0.05). **(D)** Percentage time spent in central area, calculated as a percentage of the total time spent in the OF. There was a significant interaction between WAS and DSS in modifying this parameter (*p* < 0.05).The data shown are means + s.e.m., *n* = 14–18 per group; ^χχχ^*p* < 0.001 DSS main factor effect, ^δδδ^*p* < 0.001 WAS main factor effect, ^**^p < 0.01, ^***^p < 0.001 vs. control, ^§§^*p* < 0.01, ^§§§^*p* < 0.001 vs. DSS.

A 7-day treatment with DSS had a significant influence on behavior in the OF test, which can be interpreted as an anxiogenic effect. Concomitant WAS exposure prevented the anxiogenic response to DSS treatment (Figure 3). The total traveling distance in the OF test (an index of locomotion) was significantly shortened by DSS [main factor effect: *F*_(1, 62)_ = 19.3; *p* < 0.001] and significantly prolonged by WAS [main factor effect: *F*_(1,62)_ = 28; *p* < 0.001), with no significant interaction between the factors WAS and DSS. There were significant interactions between DSS and WAS in their influence on anxiety-related parameters in the OF test, the number of central area visits [*F*_(1, 62)_ = 6.1; *p* = 0.013], percentage traveling distance in the central area [*F*_(1, 62)_ = 7.0, *p* = 0.01], and percentage time spent in the central area [*F*_(1, 62)_ = 6.3; *p* = 0.015]. *Post-hoc* analysis showed that DSS significantly increased anxiety-like behavior in the absence of WAS as deduced from a reduction of the percentage time spent in the central area and the number of central area visits (Figure 3). In contrast, in the presence of WAS, DSS failed to alter these anxiety-related behavioral parameters (Figure 3).

**Figure 4:**
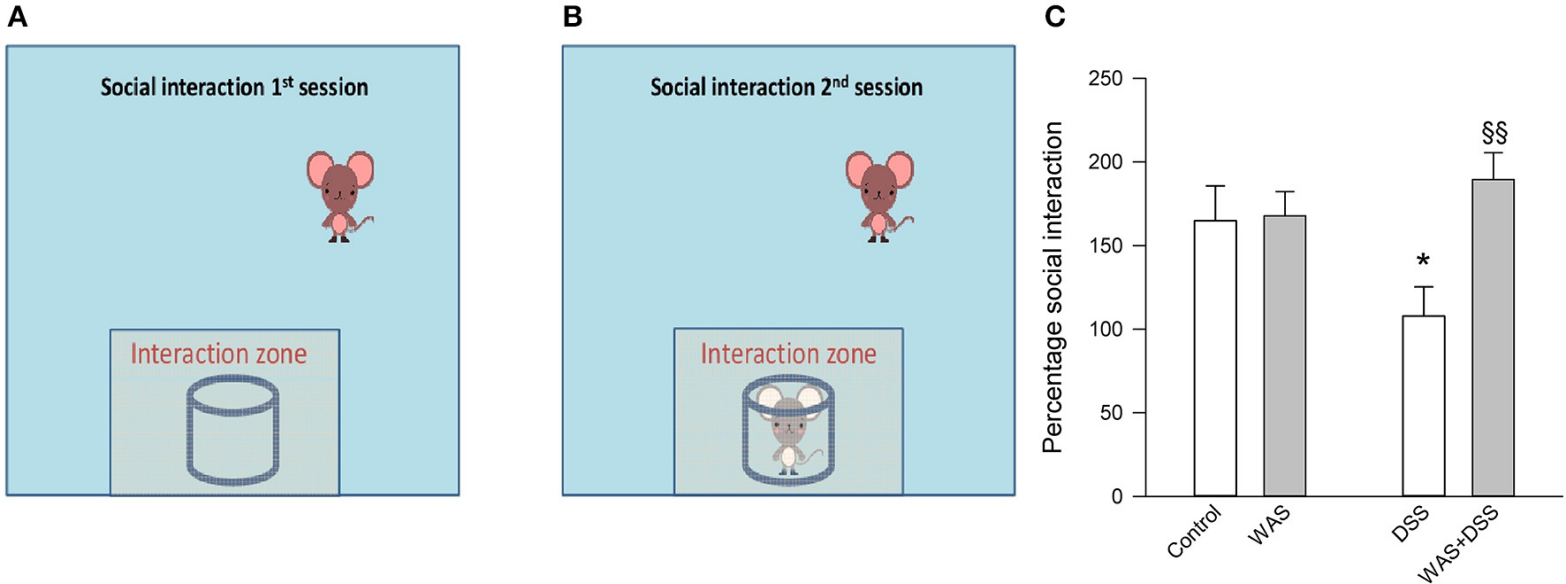
**Effect of a 7-day treatment with DSS and WAS, alone and in combination, on behavior in the SI test as carried out 2 days after the end of the treatment period**. The left **(A)** and middle **(B)** panel explain the procedure of the SI test that consisted of two sessions, the first session being conducted without a target mouse in the cylindrical container. Panel **(C)** presents the results of the SI test as percentage social interaction, calculated as the percent ratio of the time spent in the interaction zone in the presence of the target mouse divided by the time spent in the interaction zone in the absence of the target mouse. There was a significant interaction between WAS and DSS in modifying social interaction percent (*p* < 0.05). The data shown are means + s.e.m., *n* = 16–17 per group; ^*^p < 0.05 vs. control, ^§§^*p* < 0.01 vs. DSS.

In the SI test it was found that DSS treatment reduced SI and that there was a significant interaction between WAS and DSS [*F*_(1, 61)_ = 5.2; *p* = 0.027]. *Post-hoc* analysis showed that DSS significantly reduced the social activity of mice only in the absence of WAS while, in the presence of WAS, DSS did not affect social activity (Figure 4).

**Figure 5:**
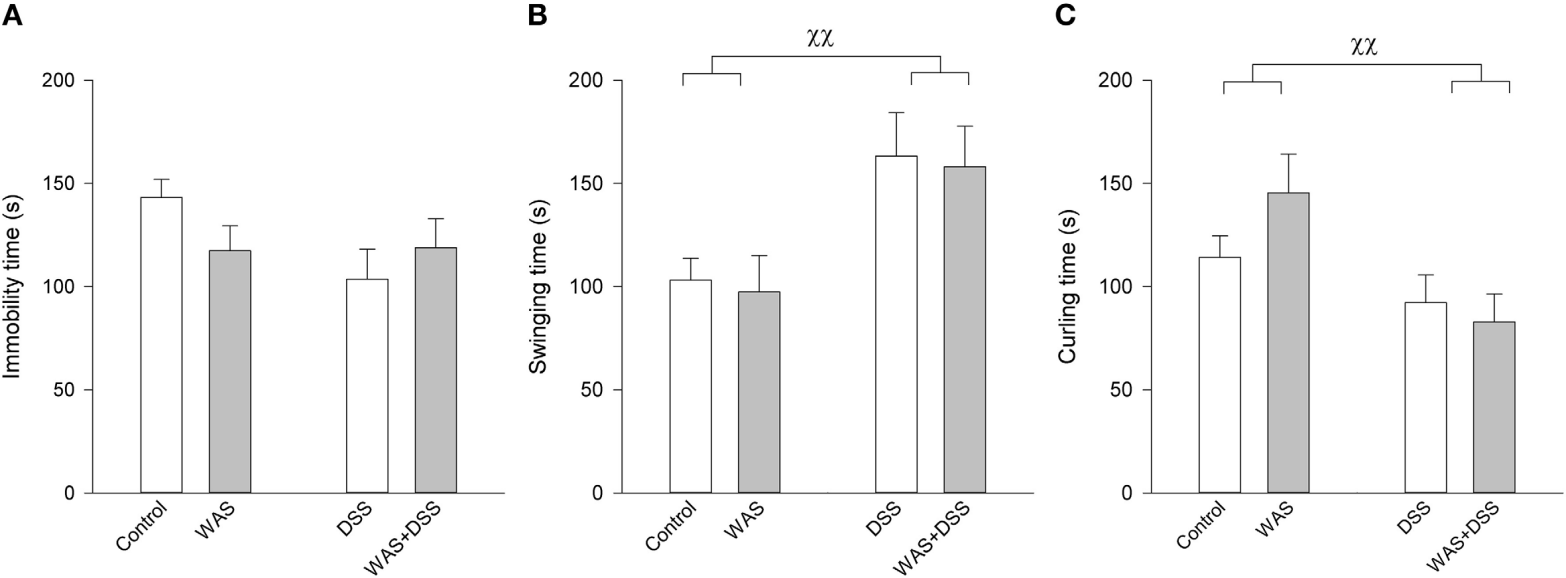
**Effect of a 7-day treatment with DSS and WAS, alone and in combination, on behavior in the TST as carried out 3 days after the end of the treatment period**. **(A)** Immobility time. Neither WAS nor DSS had a main factor effect, nor was there any interaction. **(B)** Swinging time. There was a main factor effect of DSS (*p* < 0.01) but not WAS. **(C)** Curling time. There was likewise a main factor effect of DSS (*p* < 0.01) but not WAS. The data shown are means + s.e.m., *n* = 16–17 per group, ^χχ^*p* < 0.01 DSS main factor effect.

Unlike in the OF and SI tests, a 7-day treatment with WAS and DSS, alone and in combination, had little effect on the behavior in the TST (Figure 5). Two-Way ANOVA failed to disclose any significant effect of DSS or WAS on immobility time, with no significant interaction. DSS, but not WAS, had a significant main factor effect on swinging time [*F*_(1, 61)_ = 9.7; *p* = 0.003] and curling time [*F*_(1, 61)_ = 8.6; *p* = 0.005], with no significant interaction between the two factors (Figure 5).

### DSS, but not WAS, increased plasma IL-6 and IL-18

**Figure 6:**
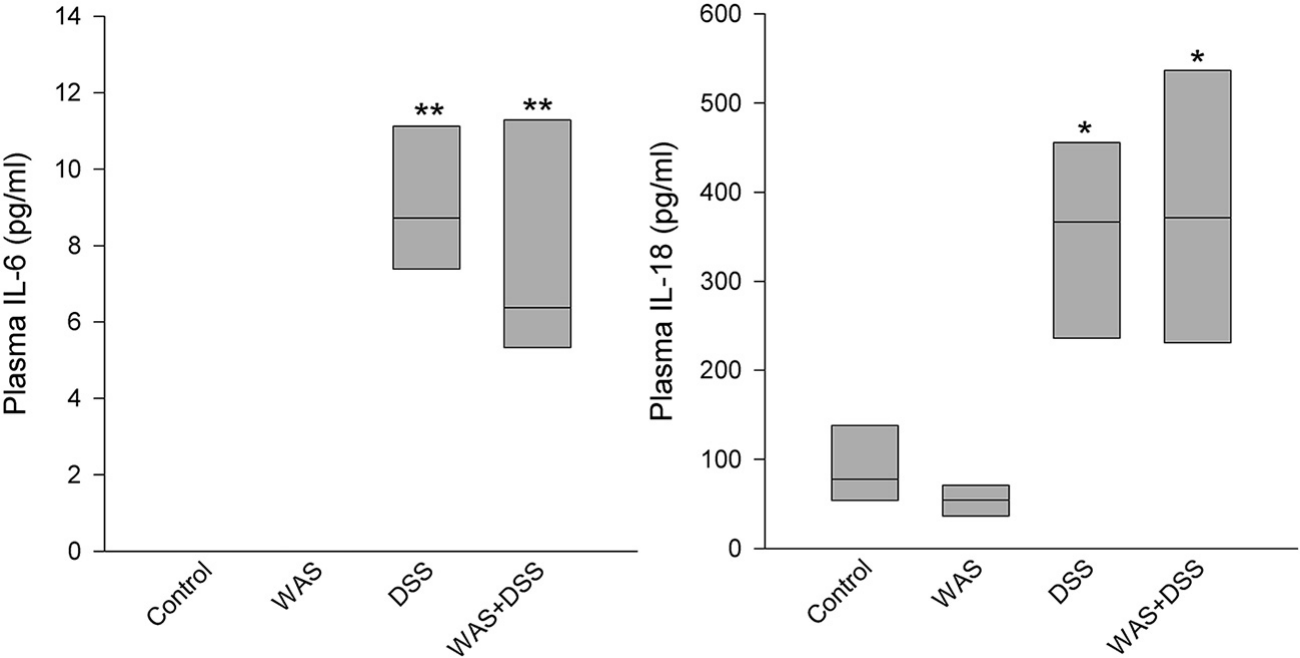
**Effect of a 7-day treatment with DSS and WAS, alone and in combination, on plasma levels of IL6 and IL18 as measured 1 day after the end of the treatment period**. The IL6 plasma levels in the control and WAS groups were below detection limit. The data shown are medians ± quartiles, *n* = 7–8 per group; ^*^p < 0.05, ^**^p < 0.01 vs. control.

Plasma levels of IL-6 were below detection limits in the control and WAS groups. The Kruskal-Wallis test revealed a significant difference among the treatment groups with regard to IL-6 (*H* = 23.9; *p* < 0.001) and IL-18 (*H* = 19.5; *p* < 0.001) (Figure 6). *Post-hoc* analysis showed that, relative to the control group, the plasma levels of both IL-6 and IL-18 were significantly increased in the DSS and WAS+DSS groups while no significant difference was detected between the control and WAS groups, on the one hand, and the DSS and WAS+DSS groups, on the other hand (Figure 6). The plasma levels of IL-10 and IL-12 were below the detection limits in all treatment groups.

### DSS increased plasma NPY independently of WAS

**Figure 7:**
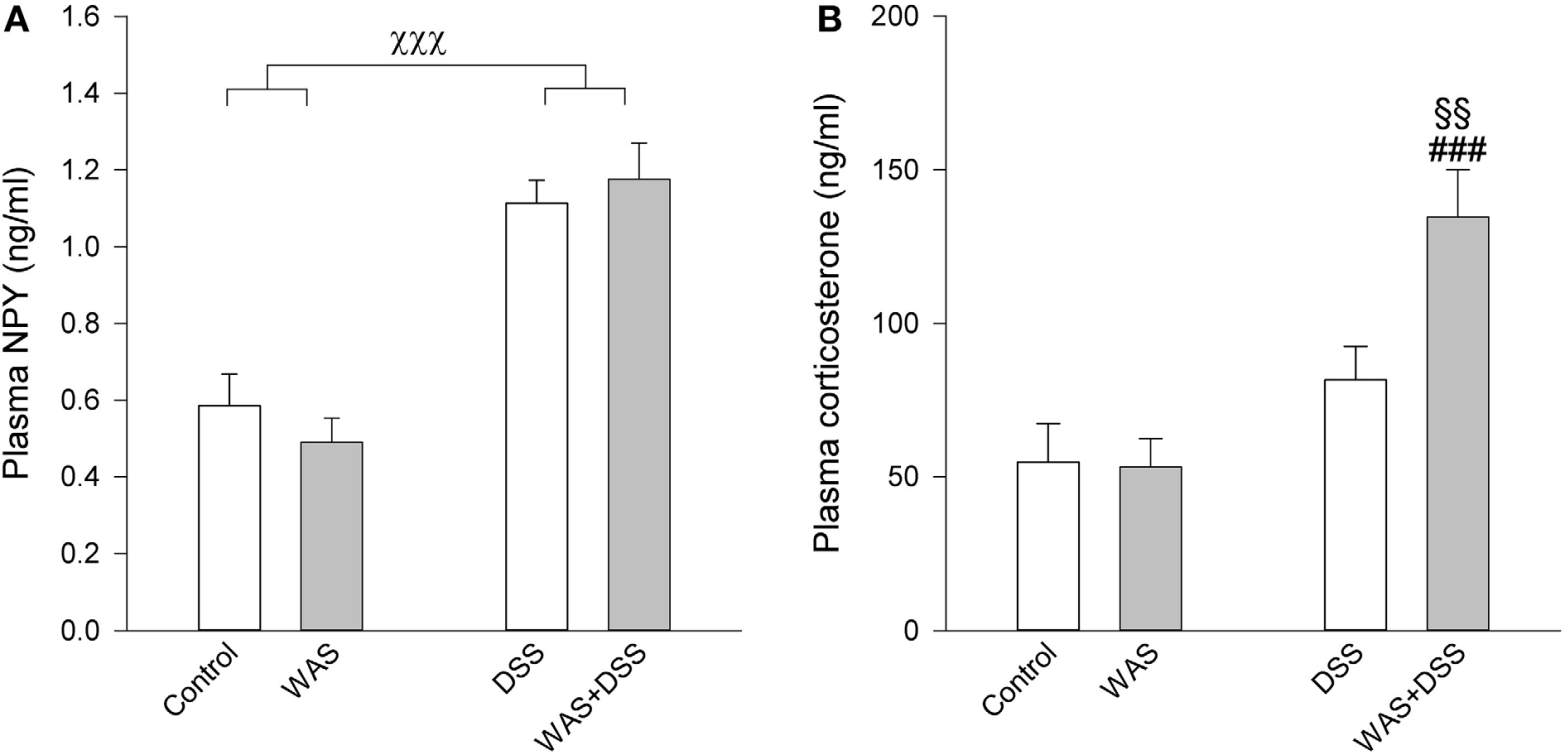
**Effect of a 7-day treatment with DSS and WAS, alone and in combination, on plasma levels of NPY (A) and corticosterone (B) as measured 1 day after the end of the treatment period**. With regard to plasma NPY, DSS but not WAS had a significant main factor effect (*p* < 0.001), with no significant interaction. As regards plasma corticosterone, there was a significant interaction between DSS and WAS (*p* < 0.05). The data shown are means + s.e.m., *n* = 7–8 per group; ^χχχ^*p* < 0.001 DSS main factor effect, ^§§^*p* < 0.01 vs. DSS, ^###^*p* < 0.001 vs. WAS.

The plasma levels of NPY were elevated by a 7-day treatment with DSS but not WAS (Figure 7A). Two-Way ANOVA revealed a significant main factor effect of DSS [*F*_(1, 27)_ = 65.5; *p* < 0.001], with no significant WAS effect and no significant interaction (Figure 7A).

### DSS increased plasma corticosterone in the presence of WAS

A 7-day treatment with DSS and WAS resulted in a significant interaction in modifying circulating corticosterone levels [*F*_(1, 27)_ = 5.1; *p* = 0.031]. *Post-hoc* analysis showed that neither WAS nor DSS had a significant effect in the absence of the other factor, while combination of the WAS and DSS treatments led to a significant increase in the plasma corticosterone levels compared to the WAS and DSS groups (Figure 7B).

### DSS treatment suppressed the relative mRNA expression of BDNF, NPY, and MR while WAS suppressed the relative mRNA expression of CRF in the hippocampus

In the hippocampus, DSS treatment blunted the relative mRNA expression of BDNF [*F*_(1, 25)_ = 15.4; *p* < 0.001], NPY [*F*_(1, 25)_ = 13.8; *p* = 0.001], and MR [*F*_(1, 25)_ = 15.4; *p* < 0.001], with no significant WAS main factor effect and no significant interaction between the two factors (Table 4). On the other hand, the relative mRNA expression of CRF was reduced in response to WAS [*F*_(1, 25)_ = 4.8; *p* = 0.035], with no significant DSS main factor effect and no significant interaction between the two factors (Table 4). The relative mRNA expression of COX-1, COX-2, and GR was not significantly changed in the hippocampus (Table 4).

**Table 4:** **Effect of DSS and WAS, alone and in combination, on the relative gene expression of various molecular factors in the hippocampus**.

**mRNA**	**Control (***n*** = **8**)**	**WAS (***n*** = **7**)**	**DSS (***n*** = **8**)**	**WAS + DSS (***n*** = **6**)**	**WAS main factor effect**	**DSS main factor effect**	**Interaction**
COX-1	1.0 (0.03)	1.0 (0.04)	0.9 (0.09)	0.9 (0.06)	NS	NS	NS
COX-2	1.0 (0.07)	1.0 (0.05)	0.9 (0.10)	1.0 (0.05)	NS	NS	NS
GR	1.0 (0.04)	1.0 (0.03)	1.0 (0.05)	0.9 (0.07)	NS	NS	NS
MR	1.0 (0.04)	1.0 (0.06)	0.8 (0.06)	0.7 (0.05)	NS	*p* < 0.001	NS
BDNF	1.0 (0.06)	1.0 (0.04)	0.8 (0.06)	0.8 (0.06)	NS	*p* < 0.001	NS
CRF	1.0 (0.10)	0.7 (0.08)	0.8 (0.10)	0.7 (0.09)	*p* < 0.05	NS	NS
NPY	1.0 (0.06)	0.9 (0.06)	0.7 (0.05)	0.8 (0.06)	NS	*p* < 0.01	NS

Animals were subjected to a 7-day treatment (days 1–7) with DSS (2% in the drinking water) and WAS (1 h daily), alone or in combination, and sacrificed for the assay of molecular factors in the microdissected brain on day 8. The data are presented as means (s.e.m.). NS, not significant.

### DSS treatment enhanced the relative mRNA expression of COX-2, while combined WAS+DSS increased the relative mRNA expression of NPY in the hypothalamus

In the hypothalamus, DSS treatment but not WAS enhanced the relative expression of COX-2 mRNA [*F*_(1, 24)_ = 18; *p* < 0.001], without any significant interaction between the two factors (Table 5). Both DSS and WAS exerted a significant interaction on the relative expression of NPY mRNA [*F*_(1, 24)_ = 8.3; *p* = 0.008]. *Post-hoc* analysis disclosed that, while WAS and DSS alone had no significant influence, combined treatment with WAS and DSS significantly increased hypothalamic NPY gene expression (Table 5). The relative mRNA expression of COX-1, GR, BDNF, and CRF was not significantly changed (Table 5).

**Table 5:** **Effect of DSS and WAS, alone and in combination, on the relative gene expression of various molecular factors in the hypothalamus**.

**mRNA**	**Control (***n*** = **7**)**	**WAS (***n*** = **7**)**	**DSS (***n*** = **8**)**	**WAS + DSS (***n*** = **6**)**	**WAS main factor effect**	**DSS main factor effect**	**Interaction**
COX-1	1.0 (0.07)	1.3 (0.28)	0.9 (0.07)	1.1 (0.13)	NS	NS	NS
COX-2	1.0 (0.18)	0.8 (0.12)	1.6 (0.13)	1.6 (0.23)	NS	*p* < 0.001	NS
GR	1.0 (0.03)	1.0 (0.03)	1.0 (0.04)	1.0 (0.09)	NS	NS	NS
MR	1.0 (0.04)	1.3 (0.20)	0.9 (0.06)	1.0 (0.10)	NS	NS	NS
BDNF	1.0 (0.05)	0.9 (0.10)	0.9 (0.06)	0.9 (0.05)	NS	NS	NS
CRF	1.0 (0.12)	1.5 (0.17)	1.2 (0.10)	1.1 (0.24)	NS	NS	NS
NPY	1.0 (0.11)	1.0 (0.15)	1.6 (0.29)	2.9 (0.31)^§§§^^###^	*p* < 0.05	*p* < 0.001	*p* < 0.01

Animals were subjected to a 7-day treatment (days 1–7) with DSS (2% in the drinking water) and WAS (1 h daily), alone or in combination, and sacrificed for the assay of molecular factors in the microdissected brain on day 8. The data are presented as means (s.e.m.).

§§§ p < 0.001 vs. DSS,

### p < 0.001 vs. WAS. NS, not significant.

### DSS treatment and WAS interact in modifying the relative mRNA expression of NPY in the amygdala

In the amygdala (Table 6), DSS treatment and WAS interacted with each other in modifying the relative expression of NPY mRNA [*F*_(1, 25)_ = 5.1; *p* = 0.033]. *Post-hoc* analysis revealed that WAS alone significantly reduced the relative NPY mRNA expression in the amygdala, while the combination of WAS and DSS abolished this effect (Table 6). The relative mRNA expression of amygdalar COX-1, COX-2, GR, MR, BDNF, and CRF was left unchanged.

**Table 6:** **Effect of DSS and WAS, alone and in combination, on the relative gene expression of various molecular factors in the amygdala**.

**mRNA**	**Control (***n*** = **8**)**	**WAS (***n*** = **7**)**	**DSS (***n*** = **8**)**	**WAS + DSS (***n*** = **6**)**	**WAS main factor effect**	**DSS main factor effect**	**Interaction**
COX-1	1.0 (0.07)	1.3 (0.28)	0.9 (0.07)	1.1 (0.13)	NS	NS	NS
COX-2	1.0 (0.13)	0.8 (0.08)	1.0 (0.09)	1.1 (0.08)	NS	NS	NS
GR	1.0 (0.03)	1.0 (0.05)	1.1 (0.03)	1.1 (0.08)	NS	NS	NS
MR	1.0 (0.04)	1.3 (0.20)	0.9 (0.06)	1.0 (0.1)	NS	NS	NS
BDNF	1.0 (0.05)	1.0 (0.08)	1.0 (0.05)	1.0 (0.05)	NS	NS	NS
CRF	1.0 (0.09)	0.9 (0.08)	1.3 (0.15)	1.1 (0.12)	NS	NS	NS
NPY	1.0 (0.06)	0.8 (0.04) ^*^	1.0 (0.09)	1.1 (0.06)^#^	NS	NS	*p* < 0.05

Animals were subjected to a 7-day treatment (days 1–7) with DSS (2% in the drinking water) and WAS (1 h daily), alone or in combination, and sacrificed for the assay of molecular factors in the microdissected brain on day 8. The data are presented as means (s.e.m.).

* p < 0.05 vs. control,

# p < 0.05 vs. WAS. NS, not significant.

## Discussion

Given that stress can be a risk factor for IBD development and relapse, the aim of this study was to investigate the interaction between repeated WAS, a psychological stress model, and DSS-induced colitis on the severity of colitis and their impact on emotional-affective behavior and potential neurochemical correlates. The key findings of our work can be summarized as follows. (i) DSS-induced colitis was associated with several behavioral changes such as reduced locomotion and enhanced anxiety-like behavior in the OF test, attenuated social activity in the SI test, and an altered escape pattern in the TST. (ii) Repeated WAS alone failed to induce any sign of colitis and had little influence on behavior, except that it enhanced locomotion. (iii) The combination of WAS and DSS treatment prevented the colitis-induced increase in anxiety-like behavior and decrease in locomotion and SI without altering the severity of colitis. (iv) DSS-induced colitis was associated with an increase in circulating IL6, IL18, and NPY, an increased expression of COX-2 mRNA in the hypothalamus, and a decreased expression of BDNF, NPY, and MR mRNA in the hippocampus. (v) The beneficial effect of WAS on DSS-evoked behavioral disturbances was associated with a significant elevation of circulating corticosterone and a significant rise in the expression of hypothalamic NPY mRNA.

### DSS-induced colitis is associated with distinct behavioral alterations

DSS-induced colitis was associated with several changes in behavior, these observations extending previous findings (Painsipp et al., 2011) and attesting to the contention that colonic inflammation impacts on brain function. Thus, DSS-induced colitis led to a reduction of locomotion in the OF test, an effect that makes it difficult to interpret the reduced time spent in, and the reduced number of visits to, the central area of the OF as indicative of an increase in anxiety-like behavior. However, previous data obtained with another strain of mice indicate that anxiety-like behavior assessed with the elevated plus maze test can be enhanced by DSS-induced colitis without a reduction of locomotion (Painsipp et al., 2011). It can hence be surmised that DSS-induced colitis is associated with reduced locomotion and attenuated curiosity/explorative behavior, which may in part be a consequence of enhanced anxiety.

In line with the clinical observation that IBD patients experience social dysfunction (Casati and Toner, 2000; Bernklev et al., 2005), we found that SI was impaired in mice with colitis. This behavioral alteration may also be a consequence of the inflammatory process, given that immune challenge and cytokine administration in rodents lead to social withdrawal (Dantzer et al., 2008). LPS, a stimulant of the innate immune system, to rats also causes a dose-dependent social withdrawal which can be blocked by inactivation of the dorsal vagal complex (Marvel et al., 2004). Although the decrease in locomotion associated with DSS-induced colitis may have an influence on the impairment of SI, the current observations nevertheless indicate that the behavioral phenotype of DSS-treated mice manifests itself in a syndrome that comprises attenuated locomotion, attenuated SI, attenuated curiosity and an apparent increase in anxiety.

DSS-induced colitis was also associated with distinct changes in escape behavior in the TST which is used to evaluate behavioral despair in an inescapable situation (Cryan et al., 2005). While the immobility time, a direct index of despair/depression-like behavior, was not altered in DSS-treated mice, the time spent swinging was prolonged and the time spent curling shortened in these mice. The inability of DSS-induced colitis to modify the immobility time in the TST is consistent with its inability to modify despair behavior in the forced swim test (Painsipp et al., 2011). Although the ethological significance of swinging and curling is not fully understood, these behavioral parameters can be differentiated in a pharmacological context (Berrocoso et al., 2013). Thus, opioid receptor agonists decrease immobility by increasing curling whereas noradrenaline and/or serotonin reuptake inhibitors decrease immobility by increasing swinging (Berrocoso et al., 2013). The distinct changes of escape behavior in DSS-treated mice will guide the future neurochemical analysis of brain function alterations arising from colonic inflammation.

### Repeated WAS fails to induce colitis and aggravate colitis severity parameters

A review of the literature shows that psychological stress paradigms have divergent effects on colitis severity. Psychological stress may increase or decrease the severity of chemically induced colitis, be devoid of any effect (Reber, 2012), or even induce mild spontaneous colitis (Reber et al., 2007).

In the current work we assessed the severity of colitis via an increase in colonic MPO content, colon weight and circulating IL-6 and IL-18 levels as well as by a decrease in colon length and body weight. As judged by these criteria, DSS treatment induced appreciable colitis, whereas WAS was devoid of such an effect. WAS also failed to interact with DSS in altering the indices of colitis severity, with the exception of colon weight which was slightly, but significantly, enhanced 1 day, but not 4 days, after the end of the 7-day treatment with WAS+DSS. However, the colitis severity parameters recorded at the two time points cannot directly be compared with each other, because the behavioral testing during days 1–3 after the end of the 7-day treatment period represents another type of stressor that may have had an effect on the colon. Despite this limitation we can conclude that repeated WAS does not induce colitis on its own and that WAS fails, by and large, to aggravate colitis severity parameters.

### Repeated WAS causes resilience to colitis-induced behavioral changes in the OF and SI tests

There is abundant literature on how stress affects brain function and behavior, and one of the major aims of this study was to examine the interaction of colitis, an internal stressor, with WAS, an external stressor, on the behavioral outcome. Repeated WAS is a validated model of psychological stress, and it has previously been reported that WAS and colitis interact with each other in signaling to, and activation of, the brain (Melgar et al., 2008; Reichmann et al., 2013). Specifically, DSS-induced colitis alters WAS-induced c-Fos expression in the prefrontal cortex, hippocampus, and amygdala (Reichmann et al., 2013). In the current work we first investigated whether these alterations in the molecular/cellular stress response manifest themselves in several behavioral domains: novelty stress in the OF test, social stress in the SI test, and inescapable stress in the TST.

Exposure of mice to WAS for 7 days affected neither anxiety-like behavior nor basal plasma corticosterone, which points to habituation as previously observed during repeated stress sessions (Luine et al., 1996; Galea et al., 1997). In addition, the ability of repeated stress to impact on anxiety may depend on animal species and strain as well as type of stressor. Thus, the effect of repeated WAS to enhance anxiety-like behavior as seen in Wistar rats (Bradesi et al., 2005) cannot be generalized because even within the same species different strains exhibit different habituation capacities (Dhabhar et al., 1997; Ryabinin et al., 1999). While we anticipated that repeated WAS would amplify the DSS-induced behavioral changes, the opposite was observed in the OF and SI tests: WAS prevented the adverse effect of DSS-induced colitis on locomotion, anxiety-like behavior and SI. The ability of WAS to counteract the effect of DSS on emotional behavior, but not that on ingestion and colitis parameters, also suggests that the anxiogenic response to DSS is unrelated to any general malaise.

Beneficial effects of predictable repeated mild stress have been reported previously (Parihar et al., 2011; Suo et al., 2013). Stress experiences do not necessarily impair health, and exposure to a moderate degree of stress that does not exhaust stress-coping mechanisms can promote future resilience to stress and in this way prevent the development of psychiatric disorders (Russo et al., 2012). The relationship between stress exposure and stress coping can be described as an inverted U shape curve relationship, wherein both low and high levels of stress impair stress coping, while intermediate levels of stress promote stress coping mechanisms (Yuen et al., 2009; McEwen and Gianaros, 2011; Russo et al., 2012).

In this context, repeated WAS for 7 days may also be considered as a model of predictable repeated mild stress, given that exposure to WAS followed a strict scheme in terms of stress duration and time of the day when the animals were subjected to stress. To the best of our knowledge, this is the first report that repeated psychological stress induces resilience to some of the behavioral manifestations of colitis. It is also important to emphasize that the beneficial effects of WAS did not extend to all adverse reactions to colitis. Thus, the DSS-evoked inflammation, anorexia, loss of body weight, and changes of escape behavior in the TST were not prevented by repeated WAS. A related observation has been made in rats subjected to chronic stress, which exhibited resilience to stress-induced cognitive dysfunction but were still susceptible to stress-induced weight loss and metabolic changes (Sweis et al., 2013). Our data are in line with the contention that stress resilience is a domain-specific rather than a global characteristic (Sweis et al., 2013).

### DSS-induced colitis is associated with distinct changes in the cerebral expression of NPY, BDNF and COX-2

The impact of DSS-induced colitis on behavioral alterations was analyzed in terms of circulating factors that may communicate between the gut and brain and cerebral factors that may explain the behavioral alterations observed. Since IBD is associated with an increased risk for anxiety and mood disorders (Walker et al., 2008; Graff et al., 2009), we focused in particular on three messenger molecules that are known for their role in the regulation of anxiety and mood: NPY, BDNF, and CRF (Holmes et al., 2003; Martinowich et al., 2007).

As alluded to before, DSS treatment for 7 days increased the circulating levels of IL-6 and IL-18, which indicates that these cytokines subserve a long-term signaling role of the inflamed colon. In contrast, the plasma levels of corticosterone were not enhanced in DSS-treated mice, which is consistent with other reports (Reber et al., 2006; Mitrovic et al., 2010) and indicates that colonic inflammation itself was devoid of a stimulant effect on the HPA axis. This conclusion is further confirmed by unaltered expression of CRF, GR, and MR in the hypothalamus of DSS-treated mice.

Another circulating factor, NPY, was significantly increased in DSS-treated mice. This observation is in line with a similar increase of plasma NPY in mice with TNBS acid-induced colitis (Baticic et al., 2011). NPY is a neuropeptide that plays a role at several levels of the gut-brain-gut axis (Holzer et al., 2012). Although other sources of NPY cannot be excluded, elevated levels of plasma NPY are likely to reflect increased release of the neuropeptide from sympathetic nerve endings (Bloom et al., 1988; Zukowska-Grojec, 1995; Renshaw and Hinson, 2001), a conclusion that is consistent with other data that colitis is associated with increased sympathetic activity (Ganguli et al., 2007; Xia et al., 2011). In addition, NPY has a role in the pathogenesis of DSS-induced colitis. Thus, NPY gene expression is increased in enteric ganglia of DSS-treated mice (Chandrasekharan et al., 2008), and deletion of the NPY gene attenuates the severity of DSS-induced colitis in mice (Chandrasekharan et al., 2008; Painsipp et al., 2011). Collectively, the rise of plasma NPY in colonic inflammation may also arise from an enhanced expression of the neuropeptide in the enteric nervous system (Holzer et al., 2012).

In the brain, NPY is one of the most abundant neuropeptides, playing a role in many brain functions such as stimulation of food intake, reduction of anxiety as well as improvement of stress coping and cognition (Heilig, 2004; Morales-Medina et al., 2010; Holzer et al., 2012). Local administration of NPY into the hippocampus causes resilience against experimental models of chronic mild stress-induced depression and posttraumatic stress disorder (Luo et al., 2008; Cohen et al., 2012). In the current work we found that the hippocampal expression of NPY was reduced in DSS-treated mice. This observation may imply that some of the behavioral alterations associated with DSS-induced colitis (e.g., enhanced anxiety and reduced SI as manifestations of impaired resilience to novelty and social stress) may be due to a hippocampal deficit of NPY. It is worth mentioning that the expression of NPY in the hypothalamus tended to increase in mice with DSS-induced colitis, which is similar to the increase in hypothalamic NPY found in mice and rats with TNBS acid-induced colitis (Ballinger et al., 2001; Baticic et al., 2011). NPY is one of the most potent orexigenic peptides, and the rise of its hypothalamic expression in colitis may reflect a counter-regulatory response to the colitis-evoked anorexia (Ballinger et al., 2001; Holzer et al., 2012).

Another effect that DSS-induced colitis had in the hypothalamus was an increase in the expression of COX-2 mRNA. COX-2 has been involved in LPS- and IL1β-induced anorexia, Asarian and Langhans (2010), which is also a symptom of IBD, of TNBS acid-induced colitis in rats and of DSS-induced colitis in mice (Gee et al., 1985; Ballinger et al., 2000; DeBoer et al., 2010). DSS-induced anorexia was also observed in the current work, and we hypothesize that this effect may be in part explained by the increase in hypothalamic COX-2 gene expression. In line with this argument is the observation that WAS neither altered food intake nor modified hypothalamic COX-2 mRNA.

BDNF has been implicated in both depression and anxiety (Duman and Monteggia, 2006). In the current work we found that hippocampal BDNF expression was significantly diminished in mice with DSS-induced colitis. Our results are in agreement with previous observations that hippocampal BDNF mRNA expression is lowered in chronic colitis induced by *Trichuris muris* (Bercik et al., 2010). This model of colitis is also associated with increased anxiety (Bercik et al., 2010), which suggests that a deficit in hippocampal BDNF may be one of the causal factors in colitis-associated behavioral perturbations.

Since hippocampal MR but not hippocampal GR decreased in response to DSS treatment, DSS treatment may lead to an imbalance in MR and GR signaling. A change in the hippocampal MR/GR ratio has been found in chronic stress-induced depression and maternally deprived infant rats and may contribute to HPA axis dysfunction and depression-like behavior (Vázquez et al., 1996; Lopez et al., 1998).

### Repeated WAS-induced resilience to colitis-related behavioral disturbances may involve the HPA axis and hypothalamic NPY

Given that repeated WAS prevented some of the behavioral perturbations in DSS-induced colitis, it was of particular interest to explore which neuronal and neuroendocrine mechanisms are specifically altered in WAS+DSS-treated mice and thus could underlie the resilience effect. One system that may be involved in the resilience effect of repeated WAS is the HPA axis. This assumption is based on the observations that combined WAS+DSS treatment for 7 days led to a significant increase in plasma corticosterone, while WAS alone or DSS treatment alone for 7 days failed to modify circulating corticosterone, although a single session of WAS activates the HPA axis (Kresse et al., 2001). A similar finding has been reported by Reber et al. (Reber et al., 2006) who showed that the diurnal plasma concentration of corticosterone remained unaffected by DSS treatment or chronic stress alone, while a combination of the two treatments elevated circulating corticosterone. This might be explained by synergistic effects of inflammatory mediators and psychological stress on the HPA axis. Inflammatory mediators are involved in the regulation of HPA axis activity, and increased proinflammatory cytokines can stimulate the HPA axis (Turnbull and Rivier, 1995). For example, IL-6 stimulates ACTH induced release of corticosterone from rat primary adrenal gland cells (Salas et al., 1990), and the cortisol response to ACTH correlates with the blood IL-6 concentration in healthy humans (Zarkovic et al., 2008). On the other hand, there is evidence that endogenous corticosterone protects from inflammation-induced behavioral changes, given that behavioral changes due to LPS, IL-1β, and viral infection are enhanced by adrenalectomy or glucocorticoid antagonists (Goujon et al., 1995; Johnson et al., 1996; Pezeshki et al., 1996; Silverman et al., 2007; Wang et al., 2011). Therefore, the increase in plasma corticosterone elicited by WAS+DSS treatment might have a role in the resilience effect of WAS against DSS-evoked anxiety-like behavior and social dysfunction, but this contention requires pharmacological confirmation.

Plasma NPY is unlikely to be involved in the beneficial effect of repeated WAS, since repeated WAS does not alter the plasma levels of NPY as found in the current project and in a previous study (Kuo et al., 2007), and circulating NPY does not differ between DSS-treated and WAS+DSS-treated mice. In contrast, the expression of NPY mRNA in the hypothalamus was significantly enhanced by the combined WAS+DSS-treatment relative to the WAS and DSS treatment alone. The resilience effect of repeated WAS is thus accompanied by an upregulation of NPY expression in the hypothalamus which attributes NPY a role in the beneficial effect of repeated WAS on the DSS-evoked behavioral disturbances. This contention is consistent with the involvement of NPY in promoting stress resilience (Cohen et al., 2012; Russo et al., 2012; Sweis et al., 2013) and with the implication of the NPY and Y receptor system in protecting from behavioral changes induced by peripheral immune challenge (Painsipp et al., 2008, 2013). In analogy with other data (Thorsell et al., 2006; Holzer et al., 2012; Sweis et al., 2013) we postulate that NPY-expressing neurons in the arcuate nucleus of the hypothalamus are of relevance to the resilience effect of repeated WAS. Neurons from the arcuate nucleus project to several brain regions implicated in the stress resilience effects of NPY including lateral septum, amygdala, periaqueductal gray and locus coeruleus (Kask et al., 2002). In addition, hypothalamic NPY may induce resilience by interacting with the HPA axis as circulating glucocorticoids can increase hypothalamic NPY, while hypothalamic NPY can stimulate the HPA axis and increase corticosterone release (Krysiak et al., 1999). However, a definite role of hypothalamic NPY in stress resilience awaits to be confirmed.

In conclusion, the present results show that experimental colitis leads to a particular range of behavioral alterations, which can be prevented by repeated WAS, a model of predictable chronic stress. The DSS-induced behavioral syndrome comprises a reduction of locomotion and exploration, an apparent increase in anxiety and a decrease in social activity and is associated with enhanced expression of COX-2 in the hypothalamus and attenuated expression of BDNF, NPY, and MR in the hippocampus. Taking account of these perturbations adds to the face and construct validity of DSS-induced colitis as a model of IBD and its association with psychiatric disorders. Although repeated WAS has little influence on behavior, it gives rise to neuroendocrine adaptations that prevent the DSS-induced alterations of locomotion, anxiety and SI to become manifest. Specifically, the resilience effect of WAS may involve adjustment of the HPA axis and upregulation of the NPY system in the hypothalamus. This interaction between an external and internal stressor sheds new light on the homeostatic mechanisms that operate under conditions of intestinal inflammation and psychological stress.

### Conflict of interest statement

The authors declare that the research was conducted in the absence of any commercial or financial relationships that could be construed as a potential conflict of interest.
